# Phylogenetic analysis of *Dichelobacter nodosus* serogroup-specific *fimA* gene from ovine footrot in Andhra Pradesh

**DOI:** 10.14202/vetworld.2015.567-571

**Published:** 2015-05-04

**Authors:** N. Vinod Kumar, A. Karthik, S. Vijayalakhsmi, D. Sreenivasulu

**Affiliations:** Department of Veterinary Microbiology, College of Veterinary Science, Sri Venkateswara Veterinary University, Tirupati - 517 502, Andhra Pradesh, India

**Keywords:** *fimA* gene, -footrot, - phylogenetic analysis, polymerase chain reaction

## Abstract

**Aim:**

Identification of different serogroups of *Dichelobacter nodosus* prevailing in the region and to understand the degree of genetic heterogeneities among the different isolates of *D. nodosus*.

**Materials and Methods:**

A total of 150 exudate samples of footrot lesions with a lesion score of 2-4 were collected from naturally infected sheep. The samples were screened by polymerase chain reaction (PCR) targeting *D. nodosus* specific 16srRNA. Of 150 samples screened, 70 samples were found to be positive. The positive samples were attempted for isolation of D. nodosus, out of which 16 isolates were recovered. All the isolates were subjected to serogrouping by multiplex PCR targeting *fimA* gene using A-I serogroup specific primers.

**Results:**

Of 16 isolates, 7 (43.75%) isolates were serogroup B, 4 (25.00%) isolates were serogroup A, 3 isolates (18.75%) were serogroup I and 2 (12.5%) isolates yielded both serogroup A and B. phylogenetic analysis was performed using neighbor-joining algorithm of the ClustelX2 software in order to study whether the serogroups isolated in the present investigation differed genetically from other published serogroups. The *fimA* gene sequence of present isolates of serogroups A, B, and I were segregated into three distinct groups with high bootstrap values. The serogroup B clustered with Australian isolate of serotype B1 suggesting high genetic similarity of the present isolate with serotype B1.

**Conclusions:**

The clinical samples were collected from suspected outbreaks of footrot and identified the prevalence of *D. nodosus* by PCR targeting 16srRNA gene. Identified serogroups A, B, and I from different districts of Andhra Pradesh. The phylogenetic analysis will help for the tentative identification of serotypes present in the serogroup and to understand the degree of genetic heterogeneities among the different isolates of *D. nodosus*.

## Introduction

Footrot is a specific contagious disease of the feet of sheep and goats, although it has been reported in cattle, horses, pigs and deer. It is an infectious syndrome caused by the synergistic action of certain bacterial species, of which *Dichelobacter nodosus* is the main transmitting agent [1].

*D. nodosus* is an anaerobic, Gram-negative, rod-shaped bacterium, with characteristic knobs at each end, and is often heavily fimbriated. The fimbriae are highly immunogenic for sheep and are the major host-protective immunogen. The current Australian classification system classified *D. Nodosus* into 10 serogroups (A-I and M) based on K-type agglutination [2]. These serogroups are further divided into 19 serotypes based on cross-absorption tests.

Currently, identification of the *D. nodosus* is being carried out by polymerase chain reaction (PCR) using 16S rRNA gene-specific primers [3]. Its serogrouping is being carried out by multiplex PCR using serogroup specific primers without the need to ­culture the organisms [4]. Despite its worldwide presence, the disease has significant economic impact in those sheep farming countries that have temperate climates and moderate to heavy rainfall, such as Australia and New Zealand. Footrot was reported as the most prevalent cause of lameness in sheep in U.K. [5]. Three different clinical forms of the disease have been described as virulent, intermediate, and benign, which are caused by the corresponding strains of the *D. Nodosus* Stewart [6]. In India, the disease has become enzootic throughout the state of Jammu and Kashmir for the last 18 years [7-9]. Similarly, outbreaks of footrot being reported regularly every year in Andhra Pradesh causing losses to the farming community. Prevalence of serogroups A, B, C, E, F and I were reported in Andhra Pradesh [10,11].

For the development of an efficacious vaccine against virulent footrot, it is essential to know the serological diversity of the *D. nodosus*. The phylogenetic analysis will help for tentative identification of serotypes present in the serogroup and to understand the degree of genetic heterogeneities among the different isolates of *D. nodosus*.

## Materials and Methods

### Ethical approval

The samples were collected from foot lesions without affecting the healthy tissues with non invasive method. Apart from this no live animal studies were taken up in the present investigation. Approval was obtained from ethical committee.

### Collection of samples

Total of 150 exudate samples of footrot lesions with a lesion score of 2-4 were collected from naturally infected sheep on sterile cotton swabs from different Districts of Andhra Pradesh. The site sampled was at interdigital space of the legs where the sensitive underlying tissues are involved.

### DNA extaction

Material collected on swabs was suspended in 100 µL of sterile phosphate buffered saline in 1.5 mL tubes by gently vortexing for 1 min. The samples were boiled for 10 min, cooled in ice for 5 min and centrifuged at 10,000 g for 1 min. Two microliters of the supernatant was used as a template for PCR reaction. Positive DNA control samples of strain JKS-05 of serogroup B (JKS05B) was obtained from Sher-e-Kashmir University of Agricultural Sciences and Technology University, Srinagar, India.

### PCR amplifiucation

PCR amplifications were performed in 25 µL volume in 0.2 mL thin-walled PCR tubes (Tarson, India). The PCR mixture contained a final concentration of 10 mM Tris-HCl, pH9.0, 50 mM KCl, 25 mM MgCl2, 0.01% gelatin, 25 p mol concentrations of each primer, 10 mM concentrations of each dNTPs and 1.0 U of Taq DNA polymerase (Bangalore Genei Pvt.Ltd., India). Oligonucleotide primers F-CGGGGTTATGTAGCTTGC and TCGGTACCGAGTATTTCTACCCAACACCT targeting 16srRNA gene were procured from Eurofins Pvt. Ltd. India). The amplification cycles in a Thermal Cycler (Kyratec, USA) was carried out as per the cycling condition described by previous workers [4]. DNA extracted from *D. nodosus* reference strain JKS05B and sterile distilled water served as positive and negative controls, respectively. The PCR products were electrophoresed in 0.8% agarose gels, stained with ethidium bromide and visualized and photographed with gel documentation system (Alpha Imager HP system, Alpha Innotech).

### Confirmation of PCR product

PCR amplicon of 16SrRNA gene (783 bp) was purified by using the QIA quick gel extraction kit (Genie spin gel extraction kit KT- 93, Genie, Bangalore) and was sequenced using the ABI 377 Perkin Elmer automated DNA sequencer at sequencing facility (MWG Biotech Pvt. Ltd, Bangalore). The reaction was carried out using 1 µg of plasmid DNA and 20 picomole of specific primers. Sequence data were analyzed using BLAST N software (http://www.ncbi.nlh.nih.gov/BLAST).

### Isolation of *D. nodosus*

For isolation of *D. nodosus*, all the swab ­samples were streaked at the place of collection on TAS agar with 4% hoof powder [12]. After inoculation, the plates were placed immediately in an anaerobic jar with Anaero Gas pack (BD, India) and carried to the laboratory. The jar was incubated at 37°C within 2-3 h. After 5 days of incubation, suspected colonies were subcultured repeatedly on the same medium containing 2% hoof powder until pure colonies of *D. ­nodosus* were obtained. Confirmation of the isolates as *D. nodosus* was done by demonstration of the typical cellular morphology in gram stained smears.

### Serogrouping of the Isolates

All the isolates of *D. nodosus* were subjected to serogrouping by multiplex PCR using A-I serogroup specific reverse primers with single forward primer [4]. The serogroup specific DNA procured from Sher-e-Kashmir University of Agricultural Sciences and Technology University, Srinagar, India was used as positive control. The serogroup M specific primer was not included in the study as the serogroup was not reported to be prevalent in the state [10]. The DNA was extracted by boiling method as described earlier by picking up 3-4 individual colonies from each isolate. The amplification conditions for multiplex PCR were similar to those of single PCR described above except for an increased concentration of the forward primer (2.5 times) as compared to reverse primers [4]. Oligonucleotide primers were procured from Eurofins Genomics India Pvt. Ltd., Bangalore. The PCR products were analyzed in 2.0% agarose gels, visualized and photographed as described above.

The PCR products were ligated into the pDrive cloning vector (Qiagen) according to the manufacturer’s recommended protocol. The ligation mixture was used to transform DH5*a Escherichia coli* cells made competent by calcium chloride as per the standard protocol. Five convergent positive colonies for each transformation were picked up and transferred into Luria Bertani broth (Hi Media, India) then incubated overnight at 37°C in a rotary shaking incubator (200 rpm). Plasmid DNA from selected colonies was extracted using a QIAprep Miniprep Kit (Qiagen, Hilden, Germany) and the concentration of the DNA adjusted to 200 ng/ml.

### Phylogenetic analysis of the Serogroups

The DNA sequencing was carried out using the ABI 377 Perkin Elmer automated DNA sequencer at sequencing facility, MWG Biotech Pvt. Ltd, Bangalore using M13 universal primers. Nucleotide sequences were assembled and aligned using Bio-Edit software program [13]. Nucleotide divergence/similarity of individual serogroups with corresponding published sequences was identified by multiple sequence analysis using MegAlign module of Lasergene package (DNASTAR Inc., USA). To construct a phylogenetic tree, the consensus nucleotide sequences of serogroup A, B, and I were trimmed manually to equivalent lengths. Phylogeny was inferred using the ­neighbor-joining algorithm of the ClustelX version, 2.0 program [14]. Bootstrapping analysis with 1000 replicates was used to estimate the robustness of the individual branches [15].

## Results and Discussion

Of 150 samples tested by PCR screening, 70 ­samples yielded the amplified product of 16srRNA gene with expected size of 783 bp. The partial sequence of 16srRNA gene found to be 100% homologous with published sequences of the corresponding gene. The sequence was submitted to the gene bank (JN008724).

All the 70 PCR positive samples when attempted for isolation of *D. nodosus* yielded 16 (22.86%) isolates. The isolates of *D. nodosus* were subjected to serogrouping by multiplex PCR using A-I serogroup specific primers. Out of 16 isolates tested, 7 (43.75%) isolates were serogroup B, 4 (25.00%) isolates were serogroup A, 3 (18.75%) isolates were of serogroup I and 2 (12.5%) isolates were showing both serogroup A and B (Table-1). This finding is in agreement with reports of previous workers [7,8] who reported serogroup B as the predominant serogroup in Kashmir.

**Table 1:** Serogroups of *D. nodosus* isolated in Andhra Pradesh.

Number of samples collected	Samples positive by 16s rRNA PCR	Number isolates obtained				

		Serogroup A	Serogroup B	Serogroup I	Serogroup A and B	Total
150	70	4 (25.00)	7 (43.75)	3 (18.75)	2 (12.50)	16 (22.86)

*Values on parenthesis are percentages, *Dichelobacter nodosus=D. nodosus*, PCR=Polymerase chain reaction

Sheep production is one of the major farm activities in the state of Andhra Pradesh and footrot has become endemic in sheep in this region [10]. The high economic significance of the disease stresses the need to explore the possibility of managing the disease by effective preventive and control measures including vaccination. Preparation of commercial inactivated vaccine containing *D. nodosus* strains present in a geographical region is a common practice. This serogroup-specific vaccination is widely used for successful control of virulent footrot in different countries including Nepal and Bhutan [16,17]. In a fimbriae based recombinant vaccine, the choice of serotype may be more critical than in whole-cell vaccines. This becomes even more important for serogroup B of *D. nodosus*, which is the most diverse serogroup, with at least six serotypes recognized [18]. Thus, for the development of an efficacious vaccine against virulent footrot, it is essential to know the serological diversity of the *D. nodosus*. In order to study whether the identified serogroups in the present investigation differed genetically from other published sequences, phylogenetic analysis was performed.

The serogroups identified in the present investigation were compared for the sequence homology with published sequences. All the partial sequences of *fimA* gene for serogroup A, B and I of present isolates were found to be identical with corresponding ­serogroups and were showing homology ranging from 97% to 99% with corresponding published sequences.

**Figure-1:**
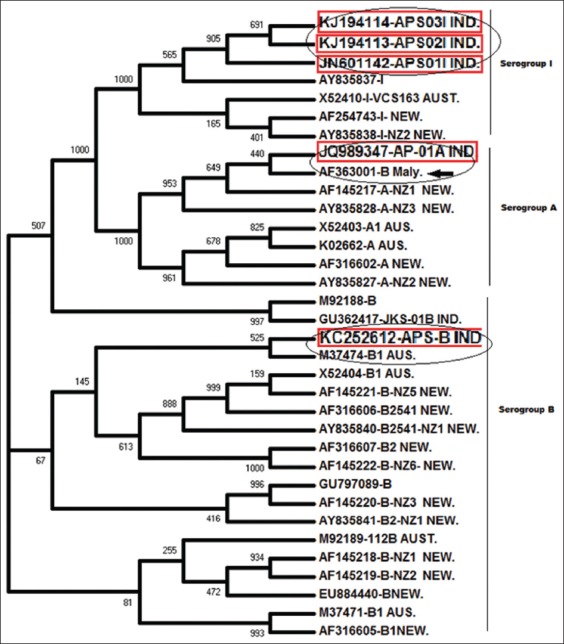
Evolutionary relationship of 34 Taxas (linearized). The evolutionary history was inferred using the neighbor-joining method. The percentage of replicate trees in which the associated taxa clustered together in the bootstrap test (1000 replicates) are shown next to the branches. The evolutionary distances were computed using the Maximum Composite Likelihood method and are in the units of the number of base substitutions per site. All positions containing gaps and missing data were eliminated from the dataset (complete deletion option). There were a total of 339 positions in the final dataset. Phylogenetic analyses were conducted in ClustelX2.

The phylogenetic tree constructed by a neighbor-joining method with fimA partial sequences of present isolates of serogroup A, B and I of *D. nodosus* were shown in the Figure 1. All the three serogroups were segregated into three distinct groups with high bootstrap values with published sequences. It was observed that, all the three serogroup I sequences of present isolates (Gen Bank Acc. No. KJ194113, KJ194114, JN601142) were clearly distinct from other closely related taxa of serogroup I (Figure-1). It was observed that the present sequence of serogroup A (JQ989347) was closely segregated with Malaysian isolate of Serogroup B (AF363001) which was clustered exceptionally in the Serogroup A. On BLAT search with GenBank, the Malaysian sequence (AF363001) was found to be showing 95-98% homology with all published sequences of Serogroup A and only 81-82% similarity with published sequences of Serogroup B, which clearly shows that this particular sequence submitted as Type B can be identified as Type A instead of Type B.

The sequence of serogroup B isolate (KC252612) was clustered with serotype B1 from Australia (M37474), suggesting the high genetic similarity of the present isolate with serotype B1. The present study on phylogenetic relatedness of isolated serogroups of *D. nodosus* with published sequences will help for the identification of genetic diversity within the serogroups. This will help in tentative identification of serotypes present in the serogroup, which will further help in better understanding of the epidemiology of ovine footrot as well as degree of genetic heterogeneities between different serogroups of *D. nodosus*.

## Conclusion

The clinical samples were collected from suspected outbreaks of footrot and identified the prevalence of *D. nodosus* by PCR targeting 16srRNA gene. The serogrouping of *D. nodosus* was done by PCR using serogroup specific primers. The isolates were identified as serogroups A, B, and I from different districts of Andhra Pradesh. Phylogenetic analysis of serogroup specific gene was done using neighbor-Joining method. Present isolates of serogroup A, B, and I were segregated in to three distinct groups. The Serogroup B sequence was closely segregated with serotype B1 of Australian isolate. Thus, the phylogenetic analysis will help for tentative identification of serotypes present in the serogroup and to ­understand the degree of genetic heterogeneities among the different isolates of *D. nodosus*.

## Authors’ Contributions

AK and SV have equally contributed in collection and processing of the samples. While NVK did laboratory analysis of the results, compiling and editing of *fim A* gene sequence for phylogenetic analysis and manuscript preparation. DS have contributed in manuscript preparation and final editing. All authors read and approved the final manuscript.
